# Squat Detection of Railway Switches and Crossings Using Wavelets and Isolation Forest

**DOI:** 10.3390/s22176357

**Published:** 2022-08-24

**Authors:** Yang Zuo, Florian Thiery, Praneeth Chandran, Johan Odelius, Matti Rantatalo

**Affiliations:** Division of Operation and Maintenance Engineering, Luleå University of Technology, 97187 Luleå, Sweden

**Keywords:** railway switch and crossing, vibration, squat, anomaly detection, unsupervised machine learning, anomaly score, point machine

## Abstract

Railway switches and crossings (S&Cs) are critical, high-value assets in railway networks. A single failure of such an asset could result in severe network disturbance and considerable economical losses. Squats are common rail surface defects of S&Cs and need to be detected and estimated at an early stage to minimise maintenance costs and increase the reliability of S&Cs. For practicality, installation of wired or wireless sensors along the S&C may not be reliable due to the risk of damages of power and signal cables or sensors. To cope with these issues, this study presents a method for collecting and processing vibration data from an accelerometer installed at the point machine to extract features related to the squat defects of the S&C. An unsupervised anomaly-detection method using the isolation forest algorithm is applied to generate anomaly scores from the features. Important features are ranked and selected. This paper describes the procedure of parameter tuning and presents the achieved anomaly scores. The results show that the proposed method is effective and that the generated anomaly scores indicate the health status of an S&C regarding squat defects.

## 1. Introduction

In recent years, rail transportation has gained significant attention due to its potential to relieve road and air congestion and environmental problems. Railway traffic in Europe has experienced a significant rise in both transporting passengers and freight in Europe [1,2]. In EU-15 countries, the passenger-kilometres and the rail freight ton-kilometres increased 28% and 15%, respectively, between 1990 and 2007 [3]. The increased volumes of freight and passenger traffic are challenges that need to be addressed because they set higher requirements on the maintenance and renewal process. To keep the railway transportation efficient, comfortable and safe under such circumstances, innovative maintenance techniques of the critical components are vital.

Railway switches and crossings (S&Cs) are important components of railway transportation infrastructure. A failure in the S&C could lead to delays globally in the system and considerable economical loss. Since S&Cs include movable parts, and are the discontinuous points of the rail geometry, they encounter high failure rates [4]. Maintaining and renewing the S&Cs across the rail network is expensive [5]. According to Cornish et al. [6], S&Cs have consumed 24% of the maintenance and 23% of the renewal budget against only 5% of the track miles in the U.K. In 2018 alone, S&Cs cost 530 MSEK, which is around 10% of the entire maintenance budget in Sweden [7]. In the worst case, such a failure could even result in catastrophic accidents due to derailments.

Due to safety concerns and their high maintenance costs, monitoring the status of S&Cs and performing preventive maintenance is important. Many studies have been performed to monitor the status of S&Cs. Most of the studies use wayside mounted systems. Liu et al. [8] experimented with two different systems. One was equipped with a 3D accelerometer and a speed detection sensor to describe crossing degradation, and the other was a video gauge system (VGS) to detect and quantify ballast conditions. However, the measurements were sensitive to the speed and the type of the train. Data from the same train type and with similar speeds were needed. Boogaard et al. [9] presented a method of utilising both accelerometers and a strain gauge mounted 50 mm below the crossing frog. Only the vibration data of the furthest measuring point from the tip of the nose were presented in the study. The results showed the advantages of combining two different measuring methods for monitoring the crossing nose. However, the proposed approach was focused on measuring the dynamics of the frog in the S&C. Barkhordari et al. [10] proposed a method of employing a wayside system to measure the track acceleration to monitor ballast degradation. However, this method does not provide continuous condition monitoring. Milosevic et al. [11] developed a condition-monitoring approach of railway crossing geometry by using measured and simulated track responses. Kerrouche A. et al. [12] proposed an experimental strain-measurement approach for monitoring the crossing nose of railway S&Cs, However, Both of these studies focused on the crossing nose instead of the whole S&C. The Axle Box Acceleration (ABA) system can also be used to monitor the status of the S&C. Wei et al. evaluated the degradation at a railway crossing using ABA measurements [13]. However, the study focused mainly on the uneven deformation between the wing rail and crossing nose and local irregularity in the longitudinal slope of the crossing nose.

Squats are one type of rail defect. According to Grosonni et al. [14], one-third of the recorded failures at the crossing panel are squat-related. Molodova et al. presented a series of studies on utilising ABA to explore the influence of different parameters and to implement an automatic squat-detection method [15,16]. However, these studies are aiming for normal tracks, and the situation for an S&C is more complicated. In addition, the ABA signal is dependent on the property of the axle box, the condition of the wheel axle bearings and the wheel profiles. Cho [17] proposed a similar method for detecting squat defects using the ABA measurement with signal processing and wavelet spectrum analysis. This study has the same drawback as the other ABA-based methods.

As critical components in railway infrastructure, S&Cs are required to be reliable in order to prevent delays and avoid fatal accidents [12]. Nowadays, manual inspection at fixed intervals is still the most commonly used way to assess the status of S&Cs [18]. These manual inspections encounter human errors and can lead to severe accidents. Manual inspection can also place inspectors in danger as regular physical access to the railway is inevitable. A plausible solution to this conundrum can be to automate the process of squat detection and monitor the health status of S&C to obtain more frequent updates of the status information, reduce the cost of inspections, reduce system down-time and increase safety. Anomaly-detection techniques are suitable for finding the segments of S&C that contain squats among the healthy data.

In most of the prior studies using wayside monitoring techniques, sensors were either installed on the side, underneath the rails or mounted on the sleeper to collect the data. These approaches of installing wired or wireless sensors are not practical due to an increased risk of damaged power and signal cables or sensors themselves under normal operation or during maintenance activities. A possible solution to overcome this issue is to make use of the protective environment within the point machines to host the sensors. This study proposed an approach of positioning the accelerometer inside the point machine to estimate the overall health condition of the S&C. The accelerometer was installed on one rod of the point machine with customised aluminum holder. This positioning provides good protection for the accelerometer against harsh weather conditions. An electrical power supply is also easily accessible from the point machine. Previously, the sensors were either installed on the axle box of the train [13,15,16,17], on the bogie of the train [19,20,21], directly on the rail [22,23] or underneath the rail [24,25]. This study proposes a new processing procedure which combines classical time-domain features with features derived from scale-averaged wavelet power (SAWP) with the help of wavelet techniques and utilises an unsupervised anomaly-detection algorithm called isolation forest to predict the anomaly score. This combination has not been yet utilised to process vibration data from the railway application. The previous studies used only time domain features [13,16,26] and supervised machine-learning algorithms [1,26]. The objective of this study is to enable continuous monitoring of the S&C to estimate its general health condition and to reduce the human interventions on track for the inspection purpose. Previous studies focused only on individual defects on normal rails [16,27,28].

The rest of the paper is organised as follows. Section 2 presents the materials and methods. Section 3 presents the results and discussions. Section 4 presents the conclusions and the future works.

## 2. Materials and Methods

The basic idea behind the current study is that the vibration at the point machine is affected by the squats on the rail head of the S&C. Squats may lead to defects, which can result in system failure during normal railway operations. The vibration is the result of a dynamic response to the wheel–rail interaction. If the rail has squats, then the vibration signal will also change its property. Therefore, analysing the vibrations can be effective in estimating the health status of the S&C.

The experiment for this study was carried out along a testbed including a full-scale S&C and a 6-tonne bogie wagon. Two levels of squats were introduced manually with 1 mm and 4 mm maximum depth. The vibration sensor is mounted at the point machine. Several signal-processing steps were applied to the original signal and 11 features were extracted for each segment of the signal. The features were the root mean square (RMS), standard deviation, shape factor, kurtosis, skewness, peak-to-peak amplitude, impulse factor, crest factor and clearance factor from time domain and the number of peaks and the total peak power from the SAWP. These features were used as input to an unsupervised anomaly-detection algorithm named isolation forest to predict if a section contained squat defects or not. By combining the results of each individual segment, the health condition of the whole S&C could be assessed. A detailed description of the methods used for this study are described in the sub-sections below.

### 2.1. Track Layout and the Testbed

**Figure 1:**
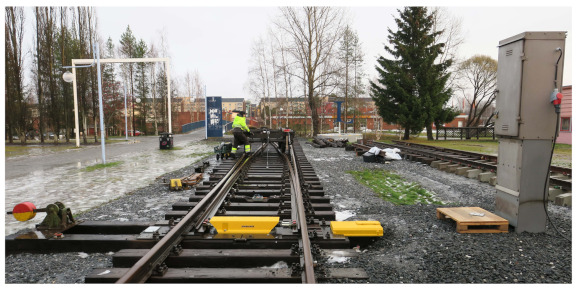
View of the test site.

**Figure 2:**
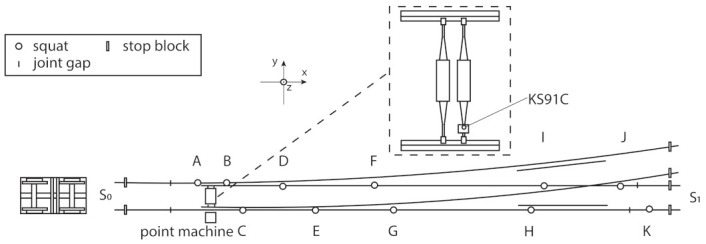
Test bed schematic diagram and accelerometer placement. The squats are labelled from A to K. $S_{0}$ and $S_{1}$ are stop blocks in the through direction.

In this study, an approach was presented to investigate how to detect and evaluate the health status of an S&C regarding squat defects by using unsupervised machine learning. The experiment was performed with a testbed located at luleå University of Technology including a full-scale S&C and a 6-tonne bogie wagon. This bogie wagon has two axles, and the distance between them is 2.5 m. The S&C used has a dimension of 1:16 and a length of 38.14 m. The accelerometer was mounted on the point machine to provide a protective environment for the accelerometer and easy access to electricity. The vibration signal and the corresponding speed information were measured. The test site is shown in Figure 1 and an illustration of the testbed is shown in Figure 2. The squats were labelled from A to K. $S_{0}$ and $S_{1}$ were two stop blocks mounted on the two ends of the rails in the through direction. The point machine is 5.86 m from the stop block $S_{0}$.

**Figure 3:**
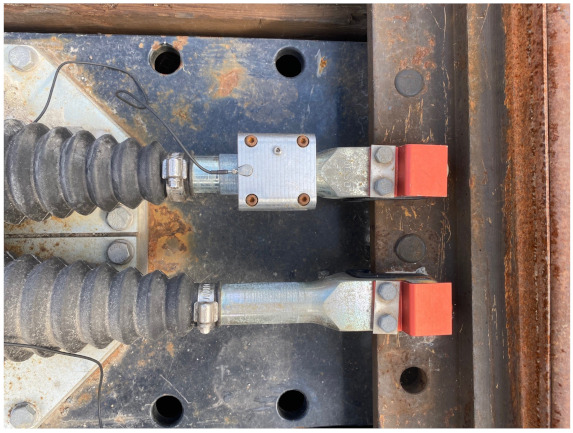
Sensor mounted on one rod of the point machine for extra protection.

To simulate two different squat levels, the squats were manually introduced stepwise with 1 mm and 4 mm maximum depths. The dimensions of the squats with two different levels were measured and are presented in Table 1. The sensor used was KS91C. It has a measuring range of 0.3–37,000 Hz, sensitivity was 10 ± 20% mV/g and the resonant frequency was greater than 60 kHz (+25 dB). The position of the accelerometer is visualised in Figure 2 and Figure 3. The vibration in the z-direction was measured. The accelerometer was glued to the aluminum holder which was mounted on one rod of the point machine.

### 2.2. Test Procedure and Data Acquisition

The experiment was performed as follows. Three different test cases were performed. The bogie wagon travelled from $S_{0}$ to $S_{1}$ without squats, with squats of 1 mm depth and with squats of 4 mm depth. Each test case was repeated 3 times. In total, 9 instances were recorded. The vibration data were measured with the accelerometer installed at the point machine. A data acquisition platform DAQ9174 was utilised to capture the vibration data and feed them to the computer directly. The sampling frequency of the platform was 51.2 kHz. The speed was measured with a customised tachometer with Hall effect senor A3144 and neodymium magnets. An Arduino Uno unit was utilised to send the revolution per minute (RPM) data of the left back wheel via WiFi to the computer. The controlling system was implemented in VI code running in LabVIEW 2019.

### 2.3. Signal-Processing Procedure

**Figure 4:**
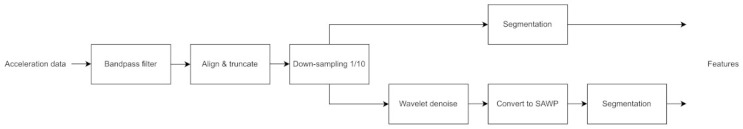
Signal-processing diagram.

**Figure 5:**
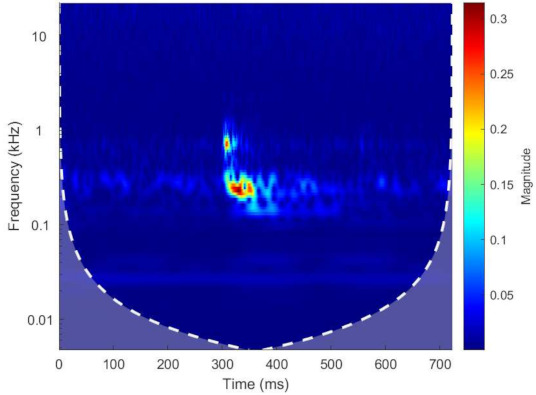
Magnitude scalogram of squat G in a 4 mm case.

The signal-processing procedure for this study is described in Figure 4. The vibration signals were initially filtered with a third-order Butterworth band-pass filter with 50 Hz and 2.5 kHz cutoff frequencies. The band-pass filter was used to filter away the frequencies with noise and preserve the useful information. A wavelet magnitude scalogram was utilised as a tool to help decide the cutoff frequency of the band-pass filter. The process is explained by using the following example. A piece of vibration data with a squat defect was extracted and evaluated with wavelet transform. Figure 5 presents the wavelet magnitude scalogram of squat G in a 4 mm case. It showed that the main energy of the response for the squat defect was around 200 Hz to 400 Hz. There was also a second frequency band around 500 Hz to 2000 Hz. This implied how the band-pass filter should be designed. The filtered signals were aligned and truncated to equal length. This step makes it possible to compare the results from different runs in the results. It could also be utilised in future studies to accurately extract the position information. Further, the signal was down-sampled to one-tenth of the original frequency. As the band-pass filter has a cutoff frequency as high as 2.5 kHz, the original signal with sampling frequency at 51.2 kHz contains redundant information. A sampling frequency at 5 kHz was enough to preserve all the useful information. To make the calculation easier, 5.12 kHz was applied. The output signals were processed in two separate paths after that. On one path, the signals were directly segmented into 400 equal-sized segments and 9 corresponding time-domain features were extracted. The features used in this study were RMS, standard deviation, shape factor, kurtosis, skewness, peak-to-peak amplitude, impulse factor, crest factor and clearance factor. On the other path, wavelet denoising was applied. The denoising was set at a level 9 decomposition, with Symlet 4 wavelet, Empirical Bayesian denoise method with median thresholding and level-dependent noise estimator. The SAWP was calculated from the output signal. Two features, the number of peaks and the total peak power, were extracted from the SAWP time series and assigned to each segment. In total, 11 features were generated. The extracted features are also described in Table 2.

### 2.4. Wavelets

The concept of wavelet transform can be traced back to 1909 when Harr introduced the first wavelet. Wavelet transform can be divided into two categories, namely, continuous wavelet transform (CWT) and discrete wavelet transform (DWT).

CWT is a very powerful tool for time-frequency analysis and can be viewed as replacing the short-time Fourier transform’s “time-frequency window” $g_{t,\xi }$ with a “time-scale window” $\Psi_{a,b}$. However, calculating all wavelet coefficients at all scales is computationally expensive, and it contains a high amount of redundant information. DWT is a good alternative in some cases. DWT works similarly to a band-pass filter and it can be performed for a signal on several levels. Each level decomposes the original signal into approximations (the low-frequency part) and details (the high-frequency part). The next level of DWT is carried out on the approximations of the previous level. Mathematically, the DWT of a function $f\left(x\right)$ is defined as the integral transform of $f\left(x\right)$ with wavelet functions $\Psi_{a,b}\left(x\right)$, when scales and positions are based on powers of two. It is defined as follows:

(1) $$DWT\left(a,b\right)=\frac{1}{\sqrt{a}}\int_{-\infty }^{+\infty }f\left(x\right)*\Psi \left(\frac{x-b}{a}\right)dx$$

where

(2) $$a=2^{j},b=k2^{j},\left(k,j\in Z^{2}\right)$$

Here *a* is called the scale factor and represents the scaling of the function, and *b* is called the shift factor and represents the temporal offset of the function. Wavelet denoising utilises DWT to decompose the original signal to obtain the wavelet coefficients, thresholding the coefficients and reconstructing the signal with reverse DWT [29]. Wavelet denoising has been widely utilised to denoise different vibration signals. Chen et al. proposed a wavelet denoising method for the vibration signals collected from wind turbines [30]. Chegini et al. [31] proposed a new application using imperial wavelet transform denoising in bearing fault diagnosis. He et al. [32] constructed a distributed acoustic sensor technology using multi-level wavelet decomposition denoising for condition monitoring of the heavy-haul railway. More details of the wavelet denoising technology implementation and application can be found in “Wavelet Denoising” by Luo and Zhang [33].

When applying wavelet denoising, a few parameters and the thresholding method needed to be decided. The maximum level of decomposition depended on signal length (N). The data acquired yielded a maximum number of decomposition levels of 21. Levels of coefficients influenced the kurtosis of the signal [34]. Increasing the number of levels of decomposition would lead to more aggressive denoising but also distort the output signal more. Empirical testing with different levels yielded 9. The wavelet function should reflect the features presented in the signal in the time domain. However, since the primary interest in this study was the SAWP time series, the different types of wavelet functions would yield the same qualitative results [35]. Symlet 4 (sym4) was chosen. There were a few methods that could be used to determine the denoising thresholds. Empirical Bayesian, block James–Stein, false discovery rate, minimax estimation, Stein’s unbiased risk estimation and universal threshold were tested. The influence on the SAWP is insignificant [35]. Since the signal without noise was not available, a quantitative comparison could not be performed in this case. Empirical Bayesian with median thresholding was chosen.

### 2.5. SAWP

The SAWP time series over scales $s_{1}$ to $s_{2}$ is defined as follows [35]:

(3) $${\bar{W}}_{n}^{2}=\frac{\delta_{j}\delta_{t}}{C_{\delta }}\sum_{j=j_{1}}^{j_{2}}\frac{{\left|W_{n}\left(s_{j}\right)\right|}^{2}}{s_{j}}$$

where

(4) $$s_{j}=s_{0}2^{j\delta_{j}},j=0,1,\ldots ,J$$

(5) $$J=\delta j^{-1}log_{2}\left(N\delta_{t}/s_{0}\right)$$

$C_{\delta }$ is scale independent and a constant for the selected wavelet function, $\delta_{j}$ is a factor for scale averaging, $\delta_{t}$ is the sampling period and $j_{1}$, …, $j_{2}$ represent scales over which the SAWP is computed. $s_{0}$ is the smallest resolvable scale and *J* determines the largest scale. $W_{n}\left(s\right)$ is the continuous wavelet transform of a discrete sequence. N is the number of points in the time series [36].

**Figure 6:**
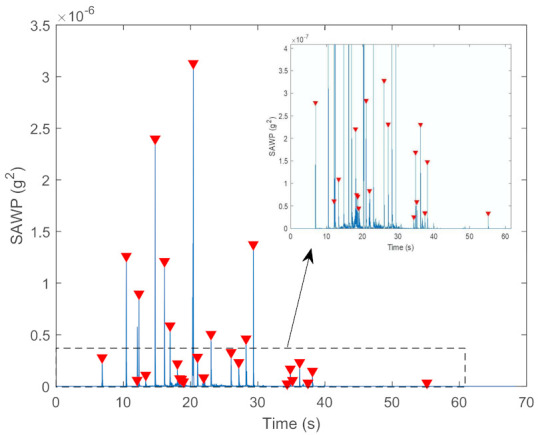
Scale-averaged wavelet power (SAWP) peaks found for a 4 mm squat case.

This can be utilised to examine fluctuations in power over a range of scales, which is exactly what was needed to detect the power burst in the vibration signal when a wheel hits a squat or a gap. This power time series will be utilised later to extract two peak-related features. The threshold for the detection of peaks was set to $2.5\times 10^{-8}\;g^{2}$. The threshold was chosen empirically. Figure 6 shows an example of the identified peaks in a 4 mm squat depth case. The corresponding number of peaks in each segment and their total power were calculated.

#### Isolation Forest

An isolation forest is an unsupervised anomaly-detection technology based on the idea of isolating anomalies instead of profiling the normal points. Given a set of observations, the isolation forest algorithm selects a random sub-sample of the observations and assigns them to a binary tree. The algorithm starts by selecting a random feature from d-dimensional features. A split is then done on a random threshold in the range of the selected feature. If the value of one observation is less than the selected threshold, it goes to the left branch; otherwise, it goes to the right. With such an approach, a node is split into left and right branches. This process continues recursively until all data points are completely isolated or when the max depth is reached. The above steps are repeated to construct random binary trees until all observations are isolated. Those points that are easier to isolate and with smaller path lengths will thus have higher anomaly scores. A comprehensive description of the isolation forest algorithm is given by Liu F.T. et al. [37].

The 11 features extracted were first scaled using normalisation. The scaled features were evaluated and selected by using both PCA and Laplacian score. After applying the isolation forest algorithm, each segment received an anomaly sore. The threshold of anomaly score to separate the healthy data and the anomalies were decided by finding the knee point. After the hyperparameters were decided, two possible indicators were proposed.

## 3. Results and Discussions

### 3.1. Segmentation

**Figure 7:**
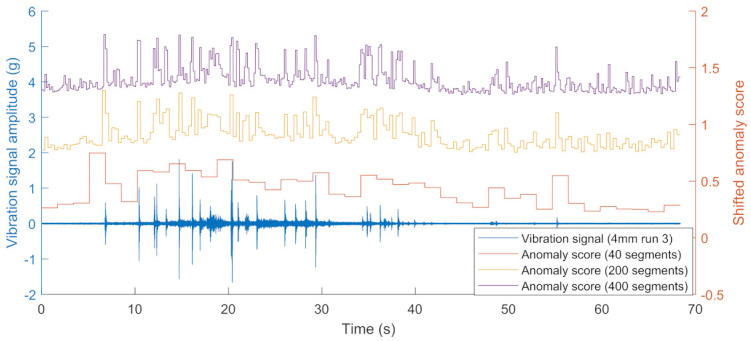
Example anomaly score for a no-squat case with different segment sizes.

All signals were aligned to have the same starting points and truncated to 350,000 samples for each signal. The signal was segmented into 20 segments and the anomaly scores achieved cannot pinpoint the precise defect location. To be able to obtain more accurate positioning of anomalies the number of segments was increased to 200 and 400, respectively. In the 400 segment case, since the speed of the bogie never exceeds 2 m/s each segment corresponds to around 0.17 s and will not be more than 0.34 m. This achieves a resolution that can be used in identifying the individual defects. The results of the influence of segment size are shown in Figure 7.

### 3.2. Feature Extraction

A total of 11 features were extracted from both the processed time-domain signal and the SAWP time series. The features can be grouped into two categories. The RMS, standard deviation, shape factor, kurtosis, skewness, peak to peak amplitude, impulse factor, crest factor and clearance factor are time-domain statistical features. The number of peaks and total peak power are extracted from the SAWP. All the extracted features are summarised in Table 2.

### 3.3. Feature Scaling

The two most used types of feature-scaling techniques are normalisation and standardisation. Normalisation is also referred to as max-min scaling, and standardisation is also referred to as Z-score normalisation. The normalisation scales the input feature values to the range of $\left[0,1\right]$, while standardisation converts the input feature values to obtain zero mean and a unit standard deviation. Since the PCA algorithm requires input features to have zero mean and a unit standard deviation, the features were standardised.

### 3.4. Feature Selection

**Figure 8:**
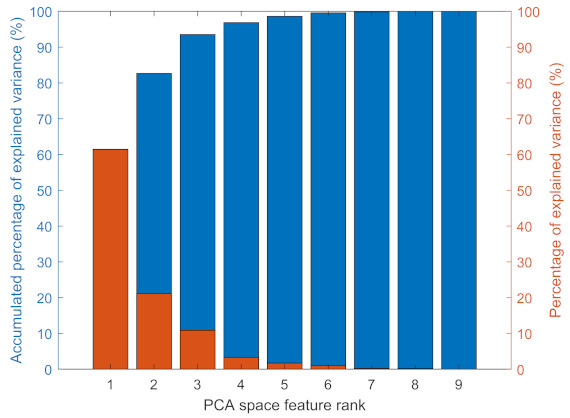
Accumulated PCA feature ranking.

**Figure 9:**
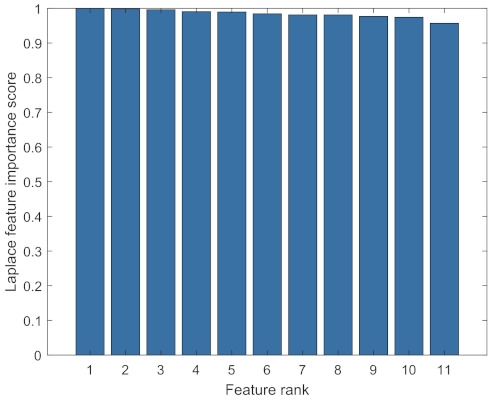
Laplacian score-based feature ranking.

Two different approaches for feature selection were utilised. The first method utilised PCA. The accumulated PCA feature importance score is presented in Figure 8. The first five features in the PCA space captured 96.55% of all the useful information. The second method employed the Laplacian score for feature selection. The redundant features were removed using the cross-correlation values between the features. Usually, the Laplacian score is defined as $L_{r}=1-s_{r}$ where $s_{r}$ is a score for each feature [38]. However, MATLAB only uses the second term $s_{r}$ which represents the feature importance. Therefore, a lower Laplacian score is equivalent to a higher feature importance score, which indicates the corresponding feature is more important. The Laplacian feature importance was calculated and ranked using MATLAB and the results are shown in Figure 9. The correlations between the most significant feature and the others are calculated. The procedures for removing correlated features are as follows. The most important feature was selected and the cross correlation between it and the rest of the features was calculated. The features that had a higher correlation value than 0.9 with the most important feature were removed. This procedure was repeated for the second most important feature in the remaining feature set. This process stopped when there were no two features left that had a cross correlation value higher than 0.9. As a result, the remaining features are features 10, 11, 9, 8, 5 and 6.

**Figure 10:**
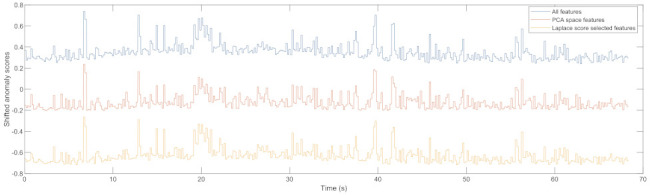
Example anomaly score for a no-squat case with different features.

The results of utilising different groups of features were compared. Figure 10 shows an example of the anomaly scores for a test case with no squat with different feature groups. The anomaly scores using PCA space features and the Laplacian score-selected features are shifted down with 0.5 and 1 correspondingly for better visualisation. The numerical comparison using mean root squared error (MRSE) is presented in Table 3. The anomaly scores generated by using all features and the PCA space features are very similar. This can be explained because the selected 5 PCA space features explain 96.55% of the variance of all features combined. The anomaly scores generated by using Laplacian score-selected features and the PCA space features are also very similar. This shows both feature selection approaches generate similar anomaly scores and are acceptable. However, the anomaly scores generated by using all features and the Laplacian score-selected features are slightly more different with around double MRSE values. The Laplacian score approach only removed five redundant features that are highly correlated with the selected most important features and the anomaly score difference is still small. Plotting and comparing the anomaly scores for those two cases verifies that the difference is so small that it is reasonable to assume similar performance.

From a performance point of view, either group of features could be utilised for further study. However, because of the curse of dimensionality, a higher dimension of features leads to exponentially increased computational efforts [39]. Therefore, it is reasonable to choose either the PCA space features or the Laplacian score-selected features. A drawback with PCA space features is that the generated features are linear combinations of the original features and they become less interpretable and lose their physical meanings [40]. Those two reasons combined justify that it is reasonable to choose Laplacian score-selected features for further study.

### 3.5. Threshold for Anomaly Score

**Figure 11:**
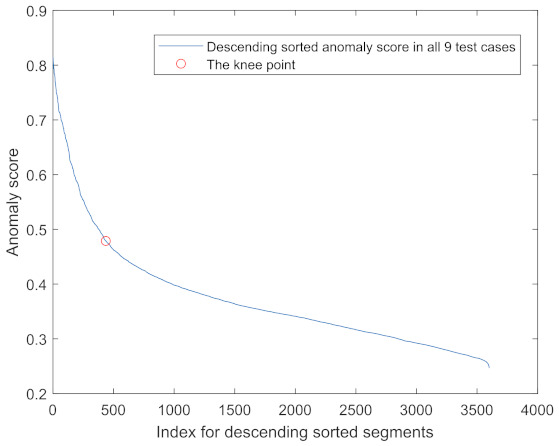
Knee point search for finding the threshold.

**Figure 12:**
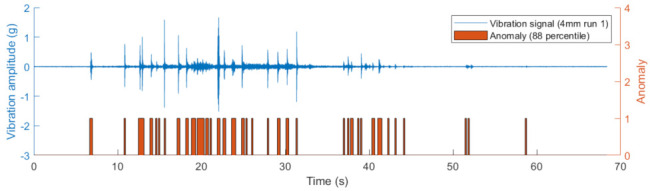
Example anomaly detection for run 1 of the 4 mm squat case with the 88th percentile as the threshold.

A threshold should be provided to decide what an anomaly is. The descend-sorted anomaly scores against the index of all 9 instances are plotted in Figure 11. Each instance contains 400 segments and these 9 instances contain 3600 segments in total. The knee point method was applied and it was found that the point with index 436 was the knee. This corresponded to around 12% of the total segments. Therefore, the 88th percentile should be used as the threshold. This is verified by plotting the anomalies using the 88 percentile together with the vibration signal. An example of the results for a 4 mm case is presented in Figure 12. By using the 88th percentile, most of the anomalies were found without introducing unexpected false alarms.

### 3.6. Anomaly Indicator for the Whole Switch

**Figure 13:**
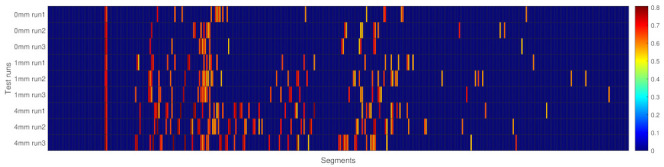
Anomaly scores above the 88th percentile threshold for all test cases.

All the test cases and the corresponding anomaly score above the threshold are presented in Figure 13. This shows clearly that with increased squat depth more anomalies are found, which indicates the health status of the S&C is degraded. It can also be observed that the different test runs were well aligned at the beginning; however, with a different speed profile for each run, the spotted defects also encounter a different drift. They are no longer well aligned after a while. This, however, would not influence the result as utilising the anomaly score as an indicator of the health status of the S&C.

One indicator could be calculating the sum of all anomaly scores and using the mean value for each test scenario. From the test data, the observed scores are 11.65, 20.31 and 29.59 for the S&C with healthy, 1 mm deep and 4 mm deep squat cases.

Another indicator could be the mean value of the number of anomalies for each test scenario. From the test data, the average number of anomalies was 18.67, 32.67 and 45.00 for the S&C with healthy, 1 mm deep and 4 mm deep squat cases.

## 4. Conclusions and Future Works

The present study demonstrates that it is possible to use the proposed method to extract features and utilise unsupervised anomaly-detection techniques, such as the isolation forest to detect the squat defects. The following conclusions can be drawn:

- The study shows that accelerometers placed within the protective environment within a point machine can be utilised for monitoring defects such as squats along the S&Cs of the railway infrastructure.
- The signal-processing procedure of extracting features from both the time domain vibration signal and the SAWP is effective and promising.
- Skewness, peak to peak amplitude, crest factor, clearance factor, Nr. of peaks and total peak power are ranked to be the top features for anomaly detection.
- The selected five PCA space features explain 96.55% of all the variance in the features.
- Anomaly-detection algorithms can be utilised to generate anomaly scores to indicate the health state of the S&C regarding squat defects. Using knee point technique, 12% of the total segments of all nine instances were determined to be anomalies.
- The mean value of the total anomaly scores for each test scenario increase from 11.65 to 20.31 and 29.59 for the S&C with healthy, 1 mm deep, and 4 mm deep squat cases. The values for 1 mm and 4 mm cases are almost 1.7 and 2.5 times greater compared to the healthy case, respectively.
- The mean value of the number of anomalies for each test scenario increases from 18.67, 32.67 and 45.00 for the S&C with healthy, 1 mm deep and 4 mm deep squat cases. The values for 1 mm and 4 mm cases are almost 1.7 and 2.5 times greater compared to the healthy case, respectively.
- An isolation forest algorithm is suitable for anomaly detection related to the squat defects.

Since isolation forest is an unsupervised machine-learning technique, no labelled data are needed to train the model. By learning from the unlabelled data, a model is built and can be utilised to perform anomaly detection on the new data. It is promising to utilise such an approach to enhance the safety and reliability of S&C. One future study would be to verify the approach with data from S&C in a real railway network. Another interesting future study could be to take into consideration such parameters as train type, load and speed among the indicators and extend the method. The future study will also include enhancing the current data set and carrying out a comparative study where the results of the proposed unsupervised anomaly-detection model will be compared to other anomaly-detection methods such as neural networks. In the future, a nationwide condition-monitoring system for S&Cs could be developed by combining such an approach and the concept of federated learning.

## Figures and Tables

**Table 1:** Dimension measurements of the two squat levels.

Squat Name	Squat Diameter 1 (mm)	Max Depth 1 (mm)	Squat Diameter 2 (mm)	Max Depth 2 (mm)
A	43	1.2	62	3.7
B	41	1.0	61	3.9
C	42	1.0	63	3.7
D	42	1.0	66	4.4
E	0	0	65	3.7
F	42	1.1	65	4.2
G	42	1.0	64	3.7
H	42	1.5	62	4.7
I	42	1.4	62	4.3
J	42	1.2	63	4.4
K	42	1.1	61	4.1

**Table 2:** Extracted features.

Feature Number	Feature Type	Feature Name
1	time domain	RMS
2	time domain	standard deviation
3	time domain	shape factor
4	time domain	kurtosis
5	time domain	skewness
6	time domain	peak to peak amplitude
7	time domain	impulse factor
8	time domain	crest factor
9	time domain	clearance factor
10	SAWP	number of peaks
11	SAWP	total peak power

**Table 3:** MRSE calculation for different feature set.

Test Run	All Features vs. PCA	All Features vs. Laplacian	PCA vs. Laplacian
	MRSE	MRSE	MRSE
0 mm_1	$8.23\times 10^{-4}$	$1.90\times 10^{-3}$	$6.12\times 10^{-4}$
0 mm_2	$8.26\times 10^{-4}$	$1.80\times 10^{-3}$	$5.13\times 10^{-4}$
0 mm_3	$1.00\times 10^{-3}$	$2.10\times 10^{-3}$	$5.24\times 10^{-4}$
1 mm_1	$7.91\times 10^{-4}$	$1.80\times 10^{-3}$	$6.26\times 10^{-4}$
1 mm_2	$8.55\times 10^{-4}$	$2.10\times 10^{-3}$	$8.40\times 10^{-4}$
1 mm_3	$8.27\times 10^{-4}$	$1.90\times 10^{-3}$	$7.28\times 10^{-4}$
4 mm_1	$7.46\times 10^{-4}$	$2.00\times 10^{-3}$	$7.59\times 10^{-4}$
4 mm_2	$8.45\times 10^{-4}$	$2.10\times 10^{-3}$	$7.91\times 10^{-4}$
4 mm_3	$8.23\times 10^{-4}$	$2.10\times 10^{-3}$	$7.63\times 10^{-4}$

## Data Availability

Not applicable.

## Sample Availability

Samples of the compounds are available from the authors.
